# Structural Validity of the Pittsburgh Sleep Quality Index in Chinese Undergraduate Students

**DOI:** 10.3389/fpsyg.2016.01126

**Published:** 2016-08-08

**Authors:** Suran Guo, Wenmei Sun, Chang Liu, Siwei Wu

**Affiliations:** ^1^Psychological and Educational Research Center, University of International RelationsBeijing, China; ^2^Institute of Education, Henan Normal UniversityXinxiang, China; ^3^Beijing Institute of EducationBeijing, China; ^4^School of Psychology, Beijing Normal UniversityBeijing, China; ^5^College of Educational Science, Hengyang Normal UniversityHengyang, China; ^6^Department of Educational and Counselling Psychology, and Special Education, University of British ColumbiaVancouver, Canada

**Keywords:** Pittsburgh Sleep Quality Index, Chinese undergraduate students, structural validity, confirmatory factor analysis

## Abstract

The purpose of this study was to examine the structural validity of the Pittsburgh Sleep Quality Index (PSQI) in Chinese undergraduate students. A cross-sectional questionnaire survey with 631 Chinese undergraduate students was conducted, and the questionnaire package included a measure of demographic characteristics, PSQI, Chinese editions of Center for Epidemiologic Studies-Depression, State- Trait Anxiety Inventory, Rumination Response Scale, and Perceived Social Support Scale. Results showed that the item “use of sleep medicine” was not suitable for use with this population, that a two-factor model provided the best fit to the data as assessed through confirmatory factor analysis, and that other indices were consistently correlated with the sleep quality but not the sleep efficiency factor.

## Introduction

Poor sleep quality is a common issue in modern society, especially among university students (Yang et al., 2003; Suen et al., 2008; Tsui and Wing, 2009). It was reported that undergraduate students suffer from sleep problems around the globe (Lund et al., 2010), as well as in China more specifically (Suen et al., 2008). In both cross-sectional and longitudinal studies, it was found that poor sleep quality significantly increased the risk for declines in social, psychological, physical, and mental health during adolescence (Roberts et al., 2008; Taylor et al., 2013). Sleep problems can trigger negative moods (anger, confusion, depression, fatigue, and tension) (Lund et al., 2010), somatic pain (Fredricksen et al., 2004), poor academic performance (Chung and Cheung, 2008), and risk taking behaviors, including drug use and drowsy driving in young adults (O’Brien and Mindell, 2005; Mollaoglu et al., 2012).

The Pittsburgh Sleep Quality Index (PSQI) (Buysse et al., 1989) is a widely used 19-item self-report questionnaire that measures subjective sleep quality, and the 19 items were transformed and combined to form seven components. This measure has showed good reliability and validity for both healthy and clinical groups with mental and physical health problems (Burkhalter and Hillman, 2011; Bush et al., 2012; Otte et al., 2013; Ho and Fong, 2014), in different age groups, from youth to elderly people (Cole et al., 2006; Curcio et al., 2013), and in different cultural contexts (Tzeng et al., 2012; Curcio et al., 2013; Hashmi et al., 2014). The Chinese version of PSQI was developed in Liu et al. (1996), widely used in adolescents, adults and olds both healthy and clinical groups, and showed good internal consistency, split half and retest reliability; and also showed good criterion related validity with age and SAS, SDS in Chinese undergraduate students (Liu et al., 1996; Qi et al., 2007; Shi et al., 2013).

However, most evidence based on the PSQI pertains to a global factor model, with total scores across all items reported as the outcome variable. It remains unclear if global PSQI scores capture all sleep attributes, particularly for undergraduate students. Cole et al. (2006) first examined the factor structure of the PSQI using a cross validation approach in a sample of 417 depressed and non-depressed individuals 60 years or older, exploratory factor analysis (EFA) showed that a two-factor model best fit the data, and confirmatory factor analysis (CFA) found that a three-factor model of sleep best fit the data.

Among the existing studies, for the two-factor model, the first factor named sleep efficiency contained two components (habitual sleep efficiency and sleep duration), and the second factor named perceived sleep quality contained other five components (subjective sleep quality, sleep latency, sleep disturbances, daytime dysfunction and use of sleep medications). In a sample of 1174 non-depressed breast cancer survivors (90% Caucasian), a two-factor model with sleep efficiency and perceived sleep quality as factors best fit the data (Otte et al., 2013). A two-factor structure was generated using EFA in a sample of 138 women with fibromyalgia (Contreras et al., 2014). Nicassio et al. (2014) also found that the two-factor model best fit data from 107 patients with rheumatoid arthritis.

On the other hand, some evidence supports a three-factor model. For the three-factor model, the first factor named sleep efficiency contained two components (habitual sleep efficiency and sleep duration), the second factor named perceived sleep quality contained three components (subjective sleep quality, sleep latency and use of sleep medications), and the third factor named daily disturbances contained two components (sleep disturbances and daytime dysfunction). Such as, in the sample of 135 Renal transplant (RTx) recipients (29% were women with a mean age of 52 years); CFA provided empirical support for a three-factor model (Burkhalter et al., 2010). Finally, a three-factor model best fit for a sample of English speaking non-Hispanic Whites and English and Spanish speaking Hispanics of Mexican descent (Tomfohr et al., 2013), as well as chronic fatigue syndrome (CFS) patients (Mariman et al., 2012).

Clearly the factor structure of the PSQI varies across studies, and this may be due to various reasons, such as different statistical methodology (CFA or EFA), sample age, country of origin, and clinical versus healthy status. And only a few studies concerned the structural factor of PSQI in undergraduate students. In a sample of 520 Nigerian university students, a three-factor model was generated using EFA (Aloba et al., 2007). In a sample of 8,481 undergraduate students from four countries, a two-factor model was found for both EFA and CFA in Chile, Ethiopia and Thailand, while a three-factor model was found for Peru (Gelaye et al., 2014). Although Chinese version of PSQI showed good reliability and validity in Chinese undergraduate students with a global score (Qi et al., 2007; Shi et al., 2013), little is known about the factor structure of the measurement in this group of people, and even in other Chinese groups. In a sample of 1145 undergraduate students and with a cutoff score of 8, 188 students (16.4%) endorsed poor sleep quality (Qi et al., 2007; Shi et al., 2013). Few undergraduate students used sleep medicine; the mean of use of sleep medication scores for 527 undergraduate students was 0.077 very low relative to other samples (Qi et al., 2007; Shi et al., 2013). The present study therefore sought to clarify which factor structure might be most applicable to this group of students, and also examined whether all PSQI items are in fact appropriate for use with this group.

In Diagnostic and Statistical Manual of Mental Disorders-IIV (DSM-5), the diagnosis of depression and anxiety included insomnia or hypersomnia these sleep quality problems. And therefore, sleep may have similar concept with depression and anxiety. The Chinese version of PSQI showed significant correlations with depression and anxiety (*r* = 0.43, 0.42) (Liu et al., 1996). The cognitive model of insomnia advanced by Harvey (2002) suggested that emotional arousal (e.g., negative emotion) and cognitive arousal (rumination and worry) before sleep can both affect sleep quality. We also examined convergent validity of PSQI with depression, anxiety, rumination and worry.

Hawkley et al. (2010) suggested that the same amount of sleep is less healthy when participants feel more socially isolated and that social support may be associated with sleep quality. So social support could influence sleep quality, but they were different concepts with sleep quality, and we examined discriminant validity of PSQI sleep quality in terms of relationships with social support.

## Materials and Methods

### Participants and Procedure

This study was a randomized sampling cross-sectional survey of undergraduate students recruited from Henan Normal University, Hengyang Normal University and Hebei Energy College of Vocation and Technology in China. The students who took part in psychology class were recruited, and the administrators were psychological teacher and undergraduate students who majored in psychology. Every administrator on the team underwent a training program prior to the survey administration to ensure quality. At the beginning of the survey, the purpose of the investigation and the nature of voluntary and confidential participant were emphasized. At last 680 questionnaires were brought back (response rate was 680/700, 97.14%). In 680 questionnaires, participants who did not complete the survey, or who left necessary variable sleep quality out, were 49 and excluded. 631 undergraduate students completed PSQI survey. And missing values for other variables were replaced by mean value.

This study was carried out in accordance with the recommendations of academic boards in Henan Normal University, University of International relations and Beijing Normal University; all participants were given written informed consent in accordance with the Declaration of Helsinki.

### Measures

#### Pittsburgh Sleep Quality Index

The PSQI is a self-report questionnaire consisting of 19 items and five additional questions (Buysse et al., 1989). The latter five questions are rated by another person such as a bed partner or roommate; these items are usually used for clinical information and so are not included in the scoring. The 19 items are combined to form seven sleep quality component scores, including subjective sleep quality, sleep latency, sleep duration, habitual sleep efficiency, sleep disturbance, sleep medication use, and daytime dysfunction. Each component score can range from 0 to 3. Item 6 and 4 was respectively transformed to from component “subjective sleep quality” and “sleep duration”; component sleep latency was formed by items 2 and 5a; habitual sleep efficiency was formed by items 1, 3, and 4; sleep disturbance was formed by items 5b–j; use sleep medication was formed by item 7; daytime dysfunction was formed by item 8 and 9. The seven component scores are summed to yield a global PSQI score ranging from 0 to 21, with a cutoff of 8 in group of Chinese people (Qi et al., 2007; Shi et al., 2013), such that higher scores indicate poorer sleep quality. The Chinese version of PSQI showed good reliability in undergraduate students (Qi et al., 2007; Shi et al., 2013). Cronbach’s α for the scale was 0.66; and composite reliability for the total PSQI was 0.81 in our study.

#### Depressive Symptoms

The Chinese edition of Center for Epidemiologic Studies-Depression (CES-D) (Wang, 1999) was used to assess depressive symptoms. It consists of 20 items each answered on a four-point scale, ranging from 0 (rarely or none of the time) to 3 (most or all of the time), with total scores ranging from 0 to 60. This scale is the most widely used depression screening scale, and the Chinese version exhibits good reliability and validity in undergraduate populations. Cronbach’s α for the scale was 0.79 in our study.

#### State Anxiety

State anxiety was assessed using the Chinese version of State- Trait Anxiety Inventory (Wang, 1999), a self-report scale. The inventory includes two subscales, and one subscale composed of the first 20 items measures state anxiety, which was examined in this study. Participants respond to each item on a scale from 1 (never) to 4 (strongly obvious). In the present study, the internal consistency Cronbach’s α was 0.89.

#### Rumination

Rumination was evaluated using the self-report version of the Rumination Response Scale (RRS), which includes 22 items. Participants respond to each item on a scale from 1 (never) to 4 (strongly obvious). This scale shows satisfactory internal consistency. In the present study, the internal consistency Cronbach’s α was 0.91.

#### Social Support

Social support was evaluated through the self-report Perceived Social Support Scale (Wang, 1999). It includes 12 items and assesses three dimensions (social support from family members, friends, and others). Each dimension includes three items, and each item is rated on a scale from 1 (strongly disagree) to 7 (strongly agree). In the present study, Cronbach’s α for the whole scale, dimensions of support from family, friends and others were respectively 0.91, 0.85, 0.88, and 0.82.

#### Other Questions

A final questionnaire contained items relating to demographic variables, including age, gender (men/women), grade, and major.

### Data Analysis

Descriptive data analyses and Pearson correlation analysis were performed using SPSS software version 22.0. Demographic data was calculated as frequencies (per cent), means, standard deviations and ranges. Person correlation analysis was conducted to test item-total correlation. All tests of hypotheses were two-tailed and the level of significance was set at *p* < 0.05.

Confirmatory factor analysis was conducted using AMOS 18.0 on the seven component scores. Through CFA, the single-factor scoring model originally proposed by the developer of the scale, a two-factor model, and a three-factor model were analyzed and compared. Multiple fit indices were used to determine adequate model fit: Comparative fit index (CFI), standard root mean square residual (SRMR), and root mean squared error of approximation (RMSEA). The cutoffs suggesting good fit are: RMSEA (0.05 < RMSEA < 0.08 indicates adequate model fit; RMSEA > 0.08 suggests poor model fit), CFI close to 1 (CFI > 0.95 indicates good fit), and SRMR value less than 0.05. The classic goodness-of-fit index chi square was also reported, and the χ^2^/df (χ^2^/df < 5 suggests adequate model fit) was relied upon to support the model fit (Hu and Bentler, 1999). Modification indices were generated by the software package; they were data-driven indicators of changes to the model that are likely to improve model fit. Factor loadings >3 were deemed acceptable.

## Results

### Descriptive Analysis

The participants included 481 females (76.2%) and 148 males (23.5%), two missing values (0.3%); 197 (31.2%) students majored in social science, 414 (65.6%) students majored in natural science or engineering, 20 missing values (3.2%); 280 students in the first year of university (44.4%), 161 students in the second year of university (25.5%), 189 students in the third and fourth year of university (29.9%), one missing value (0.2%). The mean age was 20.86 (*SD* = 1.33), with a range from 17 to 24.

Pittsburgh Sleep Quality Index global scores ranged from 0 to 14, with a mean score of 4.90 (*SD* = 2.35). Using the recommended cut-off point of 8, 14.3% (*N* = 90) of the participants reported poor sleep quality; 14.2% in the male group (*N* = 21), 14.4%. (*N* = 69) in the female group; 14.7% (*N* = 29) students majored in social science, 13.8% (*N* = 57) students majored in natural science or engineering; 13.9% students in the first year of university (*N* = 39), 13.1% students in the second year of university (*N* = 21), 15.9% students in the third and fourth year of university (*N* = 30). **Table 1** displays the descriptive statistics for the seven PSQI components. Each of the scores ranged from 0 to 3, except use of sleep medications, which ranged from 0 to 2. The lowest score was use of sleep medications at 0.01, approaching zero.

**Table 1:** Descriptive statistics and item-total correlations.

	Subjective sleep quality	Sleep latency	Sleep duration	Habitual sleep efficiency	Sleep disturbances	Use of sleep medications	Daytime disturbances	Global score
Item-total correlation	0.77 (629)^∗∗^	0.65 (629)^∗∗^	0.44 (629)^∗∗^	0.46 (629)^∗∗^	0.48 (629)^∗∗^	0.10 (629)^∗∗^	0.68 (629)^∗∗^	1
Mean	0.93	0.89	0.31	0.18	0.84	0.01	1.28	4.45
Standard deviation	0.74	0.83	0.54	0.48	0.50	0.13	0.78	2.35
Range	0–3	0–3	0–3	0–3	0–3	0–2	0–3	0–14

*^∗∗^p < 0.01*.

### Pearson Correlations

Pearson correlations were conducted among the seven components and global score (see **Table 1**). The correlations between global score and each component were all statistically significant (*p*s < 0.01), but the correlation between use of medicine and total score was the lowest.

### Confirmatory Factor Analysis

Confirmatory factor analysis was conducted to test which structure could best describe sleep quality: A one-factor model, a two-factor model consisting of a sleep efficiency factor comprised of sleep duration and habitual sleep efficiency, and sleep quality, comprised of subjective sleep quality, sleep latency, sleep disturbances, and daytime dysfunction (Gelaye et al., 2014), and a three-factor model consisting of sleep efficiency, comprised of sleep duration and habitual sleep efficiency, perceived sleep quality, comprised of subjective sleep quality, sleep latency, and sleeping medication use, and daily disturbances, comprised of sleep disturbances and daytime dysfunction (Cole et al., 2006). Before CFA, normality test was conducted with 7 components using Skewness and Kurtosis, and results were that except use of sleep medicine, the Skewness and Kurtosis of other components were all less than 1. The test of multivariate normality indicated that the data did not conform to the normal distribution, *p* < 0.001, and therefore log function was used to each component before CFA conducted.

The fit statistics for the single-factor model indicated a poor fit with the data (see **Table 2**). Standard factor loadings for use of medicine, sleep duration, and habitual sleep efficiency were all less than 0.30, which is very low (see **Table 3**). Model modification indices pointed to some sources of poor fit, and the correlation between residuals of sleep duration and habitual sleep efficiency was very strong. Even after residuals of these two components were correlated in the model, χ^2^(12, *N* = 23) = 40.32, *p* < 0.0001, χ^2^/df = 3.10; RMSEA = 0.058; SRMR = 0.0375; CFI = 0.935), standard factor loadings for those three components were still low.

**Table 2:** Fit statistics for PQSI models.

	χ^2^	χ^2^/df	RMSEA	SRMR	CFI
One factor model	40.32 (13, *N* = 22)^∗∗∗^	3.10	0.077	0.051	0.876
Two-factor model	40.31 (13, *N* = 22)^∗∗∗^	3.10	0.058	0.038	0.935
Three-factor model	34.50 (11, *N* = 24)^∗∗∗^	3.14	0.058	0.036	0.944
One factor# model	63.29 (9, *N* = 18)^∗∗∗^	7.03	0.098	0.069	0.871
Two-factor# model	37.54 (8, *N* = 19)^∗∗∗^	4.69	0.077	0.042	0.950
Three- factor# model	31.95 (6, N = 21)^∗∗∗^	5.33	0.083	0.040	0.938

*One factor^#^ model, Two-factor^#^ model, Three- factor^#^ model were models after use of medicine removed; *^∗∗∗^p <* 0.001*.

**Table 3:** Standardized factor loadings for different models.

	One factor	Two-factor	Three- factor	One factor^#^	Two-factor^#^	Three-factor ^#^
Subjective sleep quality	0.81	0.83	0.89	0.82	0.83	0.89
Sleep latency	0.42	0.42	0.40	0.45	0.41	0.40
Sleep duration	0.23	0.49	0.50	0.24	0.52	0.50
Habitual sleep efficiency	0.24	0.49	0.48	0.26	0.47	0.48
Sleep disturbances	0.36	0.36	0.39	0.36	0.35	0.39
Daytime dysfunction	0.60	0.60	0.69	0.60	0.60	0.69
Use of sleep medications	0.07	0.07	0.07	–	–	–

We also fitted the hypothesized two-factor and three-factor models, with both demonstrating good fit (see **Table 2**). Standard factor loadings for use of medicine were 0.07 for both models (see **Table 3**). Considering that the factor loading for use of medicine was less than 0.3 for each model, and the correlations between use of medicine and other components were all non-significant (see **Table 1**), so this component was deleted and confirmative factor analysis was performed again.

After use of medicine was deleted, the fit statistics for the single-factor model still indicated a poor fit with the data (see **Table 2**).

The fit statistics for the two-factor model indicated a good fit with the data; standard factor loads for each component were 0.35–0.83 and the covariance of the two factors was 0.44. The fit statistics for the three-factor model indicated poor fit with the data, χ^2^/df = 5.33, more than 5, RSMEA > 0.80; standard factor loads for each component were 0.39–0.89 and the covariance values for the three factors were 0.36, 0.41, and 0.80. And therefore, the two-factor model was accepted.

### Convergent and Discriminant Validity

The correlations among perceived sleep quality and depression, anxiety, rumination/worry, and social support were all statistically significant. However, no correlations between sleep efficiency and the other variables were significant (see **Table 4**).

**Table 4:** Pearson correlations among sleep factors and emotional, cognitive, and social factors.

	Depression	Anxiety	Rumination	Worry	Social support	Support from family	Support from friends	Support from others
Sleep efficiency	0.10	0.10	0.01	0.02	0.03	0.02	0.03	0.03
Perceived sleep quality	0.44 (629)^∗∗^	0.34 (629)^∗∗^	0.38 (629)^∗∗^	0.27 (629)^∗∗^	-0.21 (629)^∗∗^	-0.24 (629)^∗∗^	-0.16 (629)^∗∗^	-0.13 (629)^∗∗^
Global score	0.42 (629)^∗∗∗^	0.32 (629)^∗∗^	0.33 (629)^∗∗^	0.23 (629)^∗∗^	-0.16 (629)^∗∗^	-0.19 (629)^∗∗^	-0.13 (629)^∗∗^	-0.10 (629)^∗^

*^∗^p < 0.05; ^∗^*^∗^p <* 0.01; *^∗∗∗^p <* 0.001*.

## Discussion

The present study examined the factor structure of the PSQI in a sample of undergraduate students in China, using a cross-validation approach. The results showed that use of sleep medicine was not applicable to Chinese undergraduates’ sleep quality and that a two factor model appeared to best capture the structure of sleep quality for this population.

In this study, mean scores for use of sleep medicine approached zero, as did the correlations between use of medicine and total score. Factor loadings for use of medicine were very low. These results are consistent with those of Beck et al. (2004); Magee et al. (2008), Tomfohr et al. (2013), and Nicassio et al. (2014). In the study of Tomfohr et al. (2013), “sleep medications” loaded poorly and was removed from all models, and in Magee et al. (2008) the factor loadings for sleep medicine in different models were very low and all less than 0.3. Beck et al. (2004) reported low item-total correlations for the use of sleeping medication component, and Nicassio et al. (2014) removed the sleep medicine use item in each model due to low item-total correlations. These results are also consistent with the specific sleep characteristics of Chinese college students. In China, sleep problems in this population tend to be of mild or moderate severity, with these students seeking social support when such problems do occur rather than taking medication. Only 1.3% of the present sample reported regular use of sleeping medication (i.e., a score of 2 or 3 on this component).

This study provided evidence that the PSQI is a multi-dimensional measure with a sample of Chinese undergraduate students, after the use of sleep medicine item was removed. Confirmatory factor analyses revealed that a two-factor model consisting of sleep efficiency and sleep quality, along with the three factor model consisting of sleep efficiency, perceived sleep quality, daily disturbances, were favored statistically over the single factor model. This result was consistent with other studies that have examined the factor structure of the PSQI in undergraduate student samples (Aloba et al., 2007; Gelaye et al., 2014). However, the three-factor model also appeared to provide a good fit, with high covariance of perceived sleep quality and daily disturbances (0.84), such that the models were compared to identify which provides the better fit. There was no statistically significant difference between the models, so the simpler two-factor model was considered to best capture the structure of sleep quality. In the study of Gelaye et al. (2014), both EFA and CFA confirmed a two-factor model of the PSQI for undergraduates in Chile, Ethiopia, and Thailand, which was consistent with our study; however, in Aloba et al. (2007), a three factor model was generated via principal component analysis, which may be related to use of different statistical methods. Regardless, use of a two-factor model could improve the sensitivity of the PSQI when assessing sleep problems in Chinese undergraduate students.

Global PSQI scores and perceived sleep quality showed statistically significant relationships with depression, anxiety, worry/rumination, and social support. This is consistent with other studies (Bush et al., 2012; Nicassio et al., 2014). However, there were no statistically significant relationships between sleep efficiency and these other issues. In Nicassio et al. (2014), the correlation between sleep efficiency and depression was 0.063. The factor of sleep efficiency contained two components of sleep duration and habitual sleep efficiency, sleep duration was the hours of sleep, the more hours sleep and the better sleep quality was. However the diagnosis of depression and anxiety were related to insomnia or hypersomnia, sleep too short or too long could both trigger depression and anxiety, and therefore, the relation between sleep duration and other variables was not stable. The habitual sleep efficiency was the ratio of sleep hours with hours in bed, the more hours in bed and lower efficiency was. After going to bed, Chinese undergraduates typically do not fall asleep right away, often chatting with roommates, surfing the internet, or reading in bed. So the sore of efficiency was generally low and the distribution range was narrow, narrow range could lead to low correlation with other variables statistically.

In this study, sleep quality was measured using a self-report instrument, and the estimation was based on the entire scale. However, a more fine-grained assessment would be provided by use of both self-report scales and a clinical interview. And a cross-sectional design that limits causal conclusions about relationships between sleep quality and other mental health variables.

In summary, a two-factor model seems to best explain subjective sleep quality for Chinese undergraduates, with use of sleep medication essentially absent in this population. Sleep quality was associated with various indices of mental health, with no such relationships for sleep efficiency.

## Author Contributions

SG contributed study designing, data analysis, and writing; CL contributed data analysis; WS contributed sampling. SW contributed sampling.

## Conflict of Interest Statement

The authors declare that the research was conducted in the absence of any commercial or financial relationships that could be construed as a potential conflict of interest.
